# Anemia and Micronutrient Status of Women of Childbearing Age and Children 6–59 Months in the Democratic Republic of the Congo

**DOI:** 10.3390/nu8020098

**Published:** 2016-02-17

**Authors:** Sarah Harvey-Leeson, Crystal D. Karakochuk, Meaghan Hawes, Pierrot L. Tugirimana, Esto Bahizire, Pierre Z. Akilimali, Kristina D. Michaux, Larry D. Lynd, Kyly C. Whitfield, Mourad Moursi, Erick Boy, Jennifer Foley, Judy McLean, Lisa A. Houghton, Rosalind S. Gibson, Tim J. Green

**Affiliations:** 1 Food, Nutrition and Health, University of British Columbia, Vancouver, BC V6T 1Z4, Canada; sarah.harvey@ubc.ca (S.H.-L.); crystal.karakochuk@alumni.ubc.ca (C.D.K.); meaghanhawes@hotmail.com (M.H.); kristina.michaux@ubc.ca (K.D.M.); kyly@mail.ubc.ca (K.C.W.); jenniferkfoley@gmail.com (J.F.); judy.mclean@ubc.ca (J.M.); 2 Faculty of Medicine, University of Goma, Goma, Democratic Republic of the Congo; pltugirimana@gmail.com; 3 Department of Clinical Biology, College of Medicine and Heath Science, University of Rwanda, Kigali, Rwanda; 4 Faculty of Medicine, Catholic University of Bukavu, Bukavu, Democratic Republic of Congo; esto.bahizire@gmail.com; 5 Center of Research in Natural Sciences of Lwiro, Bukavu, Democratic Republic of the Congo; 6 Department of Nutrition, Kinshasa School of Public Health, University of Kinshasa, Kinshasa, Democratic Republic of the Congo; pierretulanefp@gmail.com; 7 Faculty of Pharmaceutical Sciences, University of British Columbia, Vancouver, BC V6T 1Z4, Canada; larry.lynd@ubc.ca; 8 International Food Policy Research Institute, Washington, DC 20006, USA; m.moursi@cgiar.org (M.M.); e.boy@cgiar.org (Er.B.); 9 Department of Human Nutrition, University of Otago, Dunedin 9016, New Zealand; lisa.houghton@otago.ac.nz (L.A.H.); gibson@otago.ac.nz (R.S.G.); 10 South Australian Health and Medical Research Institute, and the Women’s and Children’s Health Research Institute, Adelaide 5000, Australia

**Keywords:** anemia, deficiency, Democratic Republic of the Congo, ferritin, hemoglobin, inflammation, iron deficiency, micronutrient

## Abstract

Little is known about the micronutrient status of women and children in the Democratic Republic of the Congo, which is critical for the design of effective nutrition interventions. We recruited 744 mother-child pairs from South Kivu (SK) and Kongo Central (KC). We determined hemoglobin (Hb), serum zinc, vitamin B12, folate, ferritin, soluble transferrin receptor (sTfR), retinol binding protein (RBP), C-reactive protein, and α-1 acid glycoprotein concentrations. Anemia prevalence was determined using Hb adjusted for altitude alone and Hb adjusted for both altitude and ethnicity. Anemia prevalence was lower after Hb adjustment for altitude and ethnicity, compared to only altitude, among women (6% *vs*. 17% in SK; 10% *vs*. 32% in KC), children 6–23 months (26% *vs*. 59% in SK; 25% *vs*. 42% in KC), and children 24–59 months (14% *vs*. 35% in SK; 23% *vs*. 44% in KC), respectively. Iron deficiency was seemingly higher with sTfR as compared to inflammation-adjusted ferritin among women (18% *vs*. 4% in SK; 21% *vs*. 5% in KC), children 6–23 months (51% *vs*. 14% in SK; 74% *vs*. 10% in KC), and children 24–59 months (23% *vs*. 4% in SK; 58% *vs*. 1% in KC). Regardless of indicator, iron deficiency anemia (IDA) never exceeded 3% in women. In children, IDA reached almost 20% when sTfR was used but was only 10% with ferritin. Folate, B12, and vitamin A (RBP) deficiencies were all very low (<5%); RBP was 10% in children. The prevalence of anemia was unexpectedly low. Inflammation-adjusted zinc deficiency was high among women (52% in SK; 58% in KC), children 6–23 months (23% in SK; 20% in KC), and children 24–59 months (25% in SK; 27% in KC). The rate of biochemical zinc deficiency among Congolese women and children requires attention.

## 1. Introduction

In the Democratic Republic of the Congo (DRC), anemia is a serious health problem. According to the most recent Demographic and Health Survey (DHS) (2013–2014), the overall prevalence of anemia was ~35% among women of reproductive age (15–49 years) and ~43% among pregnant women. About 60% of children (6–59 months) were anemic, with prevalence rates over 70% in some provinces [1]. Anemia can have serious consequences for both women of reproductive age [2,3,4] and children [5,6]. Causes of anemia in the DRC include malaria [7], parasitic helminths and other infections [8], as well as sickle cell Hb [9,10]. Iron, folate, vitamin B12, vitamin A, zinc, and other micronutrient deficiencies can also contribute to anemia [11]; however, little is known about the micronutrient status of Congolese women of reproductive age and their young children [12,13]. In addition to anemia, micronutrient deficiencies can have detrimental and irreversible consequences for women and children [2,3,6]. For example, vitamin A deficiency can lead to blindness and increase mortality from infectious diseases such as measles [14]. HarvestPlus plans to introduce biofortified carotenoid enriched cassava and iron fortified beans into the DRC. Before commencing such a program, accurate prevalence estimates of micronutrient deficiencies are critical. We chose to survey two very different provinces, South Kivu (SK) in the east which is at a higher altitude and Kongo Central (KC) in the west which is a low lying area closer to the Atlantic Ocean.

The aim of this cross-sectional study was to determine the prevalence of anemia and micronutrient deficiencies among women (18–45 years) and their children (6–59 months) in SK and KC provinces in the DRC.

## 2. Methods and Materials

### 2.1. Study Location

The study was carried out in the provinces of SK and KC. SK lies in the Great Lakes area of Africa bordering Lake Kivu and Lake Tanganyika. SK borders the provinces of North Kivu, Maniema, and Katanga and shares land borders with Burundi and Rwanda. It has been an area of civil unrest for much of the last 20 years. Most of the population lives near the capital of Bukavu and at an average of 1000 m altitude or higher. SK has 34 health zones. The sampling frame was based on rural health zones that were located within a 60 km radius of Bukavu to ensure timely processing of the biochemical samples. In addition, the health zones were required to be accessible and safe to survey. Ten health zones were initially chosen, but one was dropped due to security concerns. KC is a lowland area, a narrow piece of land extending from the Atlantic Ocean to Kinshasa, the capital of the DRC. It borders the provinces of Kinshasa and Kwango, as well as the Republics of Angola, the Congo, and Cabinda. The Congo River flows through the length of the province. KC consists of five districts; the district of Lukaya was chosen for the study due to its proximity to Kinshasa and because agricultural experts and government officials thought it was representative of the wider province. The entire district, consisting of six health zones, was included in the sampling frame. For ease of reading we refer to the district of Lukaya as KC.

### 2.2. Study Design

In this cross-sectional survey, we recruited 744 mother-child pairs using a probability proportionate to size (PPS) sampling method in select health zones of SK and the rural district of Lukaya in KC, DRC between June and October 2014. Ethics approval was obtained from the Clinical Research Ethics Board at the University of British Columbia (H14-01279), the Université de Kinshasa (ESP/CE/033/14) and the Université Catholique de Bukavu (UCB/CIE/NC/25/2014).

### 2.3. Sample Size

The sample size for this study was determined using the estimates for proportion in a single cross-sectional survey. This calculation uses the estimate of the expected proportion, desired level of absolute precision, and estimated design effect to determine the number of participants required in the study. The expected proportion in this study’s estimate was based on the prevalence of anemia in children aged 6–59 months, as described in the 2013–2014 DHS: 60% in SK and 71% in KC [1]. There was insufficient reliable data on iron and vitamin A deficiency in these populations upon which to base a sample size calculation. Based on a 95% confidence level and precision of ±7.5%, we calculated the sample sizes for SK and KC to be 328 and 287, respectively. Given that the population of SK is three times larger than KC, with greater variation in geography and socio-demographic characteristics, we adjusted the sample size for SK by approximately 1.5 times to 480 (40 clusters × 12 observations). However, one health zone was excluded for security reasons, decreasing the final sample size for SK to 444 mother-child pairs. In KC, because it is recognized that good estimates of micronutrient surveys typically require at least 25 clusters with 10–40 observations per cluster [15], the sample size was increased to 25 clusters with 12 observations per cluster. This increased the final sample size in KC to 300 mother-child pairs (25 clusters × 12 observations). The total sample size for this study was determined to be 744 mother-child pairs.

### 2.4. Sampling Method

A three-stage sampling method was used to determine the study participants from within the study frame. Health areas (SK *n* = 10; KC *n* = 6) were selected from the health zones using PPS. Villages (SK *n* = 40; KC *n* = 25) were randomly selected from the health areas according to PPS. Households were selected using a “random walk” method, as village household lists were not available. At the geographical center of each village, a pen was spun. The first enumerator walked in the direction the pen pointed while two other enumerators walked in a direction 120° and 240°, respectively from that direction. The enumerators selected a household every ten houses. If the tenth household did not meet the eligibility criteria, the next house was selected until a match was found. Each enumerator continued walking until four eligible homes each were selected, for a total of 12 homes in each village. If a mother-infant pair refused participation, they were replaced with the next eligible household within the village and the identification number was reassigned.

### 2.5. Eligibility Criteria and Recruitment

A sample of 744 mother-child pairs was recruited to participate in this cross-sectional study: 444 in SK and 300 in KC. In households with more than one eligible child between 6 and 59 months old, the child was randomly selected by drawing names from a hat. Inclusion criteria for mothers were: non-pregnant women, 15–49 years, apparently healthy with no apparent chronic or debilitating condition, and who were the female heads of their household. Women were excluded if they refused to provide a blood sample, or if they refused to consent for a blood sample to be taken from their child. Lactating women were not excluded. Women completed a baseline questionnaire, which collected demographic, health, as well as household-related data.

### 2.6. Anthropometry

Anthropometric measurements were taken by trained personnel using the Food and Nutrition Technical Assistance (FANTA) Anthropometric Indicators Measurement Guide [16]. Weight and height (for children aged ≥2 years) or recumbent length were measured for each child and mothers wearing light clothing and no shoes. Mid-upper arm circumference (MUAC) was measured for children only. Duplicate measurements were taken using standardized techniques and calibrated equipment [17]; a third measurement was obtained if the difference between the first two measurements was outside the allowable difference for that measure [18]. Children’s anthropometric z-scores were calculated using a plug-in for World Health Organization (WHO) Anthro (WHO, Geneva, version 3.2.2) for Stata software version 13.1 for Mac (Stata Corp, College Station, TX, USA). Extreme outliers were excluded as follows: (WAZ) ≤ −6 or ≥5, weight-for-height Z score (WHZ) ≤ −5 or ≥5, height-for-age Z score (HAZ) ≤ −6 or ≥6, and mid-upper arm circumference Z score (MUACZ) ≤ −5 or ≥5.

### 2.7. Blood Collection, Processing, and Analysis

Women and children were non-fasting and blood was collected in the afternoon. Blood was collected by venipuncture into 8 mL trace-element free evacuated tubes (BD Vacutainer^®^) that contained no anti-coagulant. Two drops of blood were removed from the tube for measurement of Hb. Hb concentration was determined by finger prick using a Hemocue^®^ (Hemocue 301+, Angelholm, Sweden). The remaining blood was allowed to clot at room temperature for 30 min and then placed on ice for transport to the hospital for processing between 2 and 4 h. Serum samples were separated using trace-element free techniques and frozen at −80 °C prior to shipment on dry ice for subsequent analyses. Serum samples were analyzed for ferritin, soluble transferrin receptor (sTfR), retinol binding protein (RBP), C-reactive protein (CRP), α-1 acid glycoprotein (AGP), zinc, vitamin B12, and folate concentrations. A complete blood count was conducted on a subset of participants (*n* = 104 children, *n* = 105 women), from both regions, using an automated hematology analyzer (Sysmex XT-1800i, Japan). Serum ferritin, sTfR, RBP, CRP, and AGP were measured using a combined sandwich enzyme-linked immunosorbent assay [19]. Serum zinc was analyzed by flame atomic absorption spectrophotometry (AAS) using a modified method of Smith *et al*. [20]. Serum folate was measured using the microbiological assay as described by Molloy and Scott [21]. Vitamin B12 was analyzed using the Roche Elecsys^®^ 2010 automated analyzer and serum retinol (ROH) was measured using HPLC [22]. Serum ROH was analyzed in a sub-sample for comparability to RBP as an indicator of vitamin A status. Quality control checks were conducted and CVs were found to be within recommended parameters for each biochemical indicator. The calculated CVs for ferritin, RBP, sTfR, CRP, AGP, zinc, and vitamin B12 were 2.9%, 3.3%, 3.3%, 6.7%, 9.2%, 5.2%, and 9.8%, respectively. The CVs for the folate assay, based on low, medium, and high reference ranges, were 14.6%, 11.9%, and 13.8%, respectively. Capillary blood was collected using a finger prick for a rapid malaria test (CareStart, AccessBio Inc., Sommerset, NJ, USA). This diagnostic test could detect all four Plasmodium species.

### 2.8. Data Preparation and Statistical Analysis

Hb concentrations were adjusted downwards for altitude (according to altitude level at the village level based on GPS) for all women and children living in villages with an altitude above 1000 m. For example, for those living in villages between 1000 and 1250 m, Hb concentration was adjusted downwards by 2 g/L. Adjustment values increase with altitude level (based on a 10-level scale) [23]. Hb concentrations were adjusted for ethnicity at the individual level by adjusting upwards by 10 g/L for all women and children regardless of age (as all women and children in our study were of African origin) as proposed by the UNICEF/UNU/WHO [23].

Ferritin and RBP concentrations were adjusted for inflammation using biomarkers (CRP and AGP) according to methods proposed by Thurnham *et al*. [24,25]. Thurnham’s correction factors based on the three stages of inflammation (incubation (CRP > 5 mg/L), early convalescence (CRP > 5 mg/L and AGP > 1 g/L), and late convalescence (AGP > 1 g/L)) for ferritin are 0.77, 0.53 and 0.75 [24]; and for RBP are 1.13, 1.24 and 1.11 [25], respectively. Zinc was adjusted for inflammation using study-generated correction factors, which were determined with linear regression using the natural log of serum zinc concentration; and correction factors were calculated by 1 divided by the geometric mean ratio, according to the three stages of inflammation. The study-generated correction factors based on the three stages of inflammation for zinc were 1.01, 1.15 and 1.07 for children, and 1.07, 1.09 and 1.08 for women, respectively. Iron deficiency anemia (IDA) prevalence estimates were based on low Hb values that were adjusted for both altitude and ethnicity. IDA based on elevated sTfR (>8.3 mg/L) is indicative of tissue iron deficiency, whereas IDA based on low ferritin concentrations (<15 µg/L for women; <12 µg/L for young children) is indicative of storage iron depletion in the absence of inflammation. Mean ± SEM and prevalence estimates (95%CI) are presented [26].

Data analysis was conducted using Stata version 13.1 for Mac (Stata Corp, College Station, TX, USA). *T*-tests and chi-square tests were used to examine differences between groups. Two-sided *p* values < 0.05 were used to determine statistical significance.

## 3. Results

### 3.1. Household Characteristics

Refusal rates were 13% for SK and 3% for KC. Household characteristics for the mother-child pairs (*n* = 744) are summarized in Table 1. Households in SK were larger than KC, with a mean ± SD size of 6.6 ± 2.6 members in SK, compared with 5.7 ± 2.3 in KC. Women in KC had higher reported levels of completed education. The majority of women in SK received no schooling (48%), whereas the majority of women in KC had received primary school education (47%). Almost 40% of women in both provinces were lactating. The majority of households in SK had a metal/tin roof (69%), whereas the majority of households in KC had a leaf roof (62%). Both SK and KC provinces reported charcoal as the main source of household fuel (73% and 64%) and pit latrines as the type of household toilet (95% and 80%, respectively). Children in SK and KC provinces received similar health services, with children receiving a vitamin A capsule (89% and 93%) and a deworming tablet (83% and 77%, respectively) in the last six months.

### 3.2. Anemia, Micronutrient, and Infection Status

Mean age and concentrations of micronutrients, Hb, and inflammation biomarkers among women of childbearing age by province are presented in Table 2 and among children 6–59 months by province in Table 3.

Body mass index and prevalence of micronutrient deficiencies, anemia, storage iron depletion (ferritin), tissue iron deficiency (sTfR), inflammation (CRP and AGP), and malaria among women of reproductive age by province are presented in Table 4 and among children 6–59 months by province in Table 5. The majority of women had a normal body mass index (BMI) (18.5–24.9 kg/m^2^) in both SK and KC provinces (74% and 76%, respectively). There was a smaller proportion of underweight women (3% *vs.* 15%) and a higher proportion of overweight women (21% *vs.* 7%) in SK as compared to KC, respectively.

Overall, there was a high prevalence of zinc deficiency among women and children that did not statistically differ by province. The prevalence of zinc deficiency (adjusted for inflammation) was high among women (52% in SK; 58% in KC), children 6–23 months (23% in SK; 20% in KC), and children 24–59 months (25% in SK; 27% in KC). Serum zinc concentrations were adjusted for inflammation using study-generated correction factors determined based on the three levels of inflammation using CRP and AGP concentrations: incubation (CRP > 5 mg/L), early convalescence (CRP > 5 mg/L and AGP > 1 g/L) and late convalescence (AGP > 1 g/L). Adjustment for inflammation decreased the prevalence of zinc deficiency in all population groups: only slightly among women (from 55% to 52% in SK; 60% to 58% in KC), but greater among children 6–23 months (from 36% to 23% in SK; 31% to 20% in KC), and children 24–59 months (from 35% to 25% in SK; 39% to 27% in KC).

Acute (CRP > 5 mg/L) and chronic (AGP > 1 g/L) inflammation were more prevalent in KC as compared to SK province. The prevalence of chronic inflammation was higher in children (ranging from 61% to 71% by province) as compared to women (ranging from 23% to 24% by province).

The prevalence rates of folate and B12 deficiency were low (all < 5%) among women and children. RBP concentrations (as an indicator of vitamin A status) were higher in KC, as compared to SK. We analyzed ROH on a sub-sample of mothers and children, for comparability to RBP as an indicator of vitamin A status. Among mothers (*n* = 48), Pearson’s correlation between RBP and ROH concentrations was *r* = 0.59 and the concordance correlation was 0.55 (95%CI: 0.33, 0.71). The concordance plot showed that RBP concentrations were overall slightly higher than ROH concentrations (mean difference = 0.17). Among children (*n* = 50), Pearson’s correlation between RBP and ROH concentrations was 0.52 and the concordance correlation was 0.49 (95%CI: 0.29, 0.69). The concordance plot did not show any trends between the two measurements (mean difference = 0.04). Further, inflammation-adjusted serum RBP and ROH concentrations showed overall similar prevalence rates of vitamin A deficiency among the *n* = 48 women (<1% in both SK and KC) and *n* = 50 children 6–59 months (both < 1% in SK; 4% *vs.* 17% in KC) in our study.

The prevalence of iron deficiency was seemingly higher with sTfR than with ferritin among women (18% *vs.* 4% in SK; 21% *vs.* 5% in KC), children 6–23 months (51% *vs.* 14% in SK; 74% *vs.* 10% in KC), and children 24–59 months (23% *vs.* 4% in SK; 58% *vs.* 1% in KC). A lower proportion of children without inflammation had an elevated sTfR (*n* = 69; 31%), as compared to children with incubation (CRP > 5 mg/L; *n* = 6; 55%), early convalescence (CRP > 5 mg/L and AGP > 1 g/L; *n* = 114; 61%) and late convalescence (AGP > 1 g/L; *n* = 130; 48%). Examining children with or without inflammation based on any inflammation stage, a significantly higher proportion of children with an elevated sTfR was observed among children with any elevated inflammation biomarker (*n* = 250; 78%), as compared to children with no inflammation (*n* = 69; 22%, chi-square test *p* < 0.0001). A lower proportion of women without inflammation had elevated sTfR (*n* = 90; 16%), as compared to women with incubation (*n* = 4; 29%), early convalescence (*n* = 8; 19%), and late convalescence (*n* = 38; 30%). When we looked at women with elevated sTfR (*n* = 140) with or without inflammation based on any inflammation stage, a lower proportion of women with elevated sTfR was observed among women with any elevated inflammation biomarker (*n* = 50; 36%), as compared to those women with no detectable inflammation (*n* = 90; 64%, chi-square test *p* < 0.001).

Malaria prevalence significantly differed by province, with higher rates in KC than in SK. Among women, the prevalence of malaria was 7% in KC and 2% in SK, whereas among children, the prevalence among children 6–23 months was 34% in KC and 2% in SK, and among 24–59 months was 44% in KC and 4% in SK.

Anemia prevalence (without adjustment) among women was only 10% in SK but nearly 30% in KC. Adjustment for altitude increased anemia rates by 6% in SK but had a minimal effect in KC. Adjustment for ethnicity lowered anemia rates to 4% and 9% in SK and KC, respectively. Adjustment for ethnicity and altitude did not alter the prevalence of anemia appreciably as compared to adjustment for ethnicity alone. Anemia prevalence was similar in both provinces (~40%) for children 6–23 months. However, for children 24–59 months anemia prevalence remained at ~40% in KC but dropped to 23% in SK. Adjustment for altitude and ethnicity in children showed a similar pattern to mothers. Mean corpuscular volume (MCV) was measured in a sub-sample of women (*n* = 105) and children (*n* = 104) from both regions. Among those anemic women (*n* = 21; using altitude adjusted Hb), the majority (57%) had normocytic (MCV 80–95 fL), 33% had microcytic (MCV < 80 fL), and 10% had macrocytic anemia (MCV > 95 fL) [27]. Among the children with anemia (*n* = 39; using altitude adjusted Hb), the majority (56%) had microcytic (MCV < 77 fL, for children 6–35 months and < 79 fL for children 36–59 months), 31% had normocytic (MCV 77–86 fL for children 6–35 months and 79–86 fL for children 36–59 months), and 13% had macrocytic anemia (MCV > 86 fL). IDA among women was seemingly low (<3%) regardless of whether ferritin or sTfR was used as the indicator of iron deficiency. IDA was low based on serum ferritin in children, except in children 6–23 months in SK where it was almost 10%. In contrast, IDA based on sTfR was 15%–20% except in children 24–59 months where it was only 50%

### 3.3. Children’s Anthropometry

The proportion of children that were underweight, wasted, stunted, and with low MUAC (defined as <−2 SD) are presented in Table 6. The proportion of underweight children (WHZ < −2 SD) was higher in SK (26%), as compared to KC (14%); however, this difference was not observed across provinces for children 24–59 months (26%). Wasting (WHZ < −2 SD) was highest among children 6–23 months in KC (7%) and lowest in children 24–59 months in the same province (4%). Overall, stunting prevalence (HAZ < −2 SD) was high among children 6–23 months (39%) and 24–59 months (62%), regardless of province. Stunting was highest among children 24–59 months in SK (67%) and lowest in children 6–23 months in KC (30%).

## 4. Discussion

Here we report our comprehensive assessment of micronutrient status of women of childbearing age and children <5 years in SK and KC provinces in the DRC. Anemia prevalence rates varied widely according to adjustments for altitude and ethnicity, as compared to adjusting only for altitude. Anemia cutoffs should be adjusted upwards when a population lives at 1000 m or higher above sea level [28]. At higher altitudes, there is a reduction in oxygen saturation in the blood (hypoxia), which stimulates Hb production resulting in higher Hb concentrations [29]. Individuals of African ethnicity have been shown to have lower Hb concentrations than other ethnic groups, regardless of iron status [30,31,32]. The WHO have proposed that individual level Hb concentrations be adjusted upwards by 10 g/L for all individuals of African origin regardless of age [33]. However, there is controversy around this proposed recommendation: some evidence exists to support a lower Hb cut-off for individuals of African origin [30,34]; while the most recent (2008) global recommendations summarized by Sullivan *et al*. [28] advocate for Hb adjustment only for age, sex, pregnancy status, altitude, and cigarette smoking [23,28,35]. Based on these classifications of anemia [23], this changed the prevalence of anemia from a severe (>40%) to a moderate public health problem (20%–40%) in three of our six population groups, which could have substantial implications for nutrition policy and programming. Anemia prevalence in our study was much lower than the national prevalence rates reported by the 2013–2014 DHS [1], regardless of Hb adjustments. Hb concentrations can vary by method of measurement and sample collection [36], which could be contributing to some of the differences observed. Given that most of the women and children with anemia had only mild anemia, a minor systematic change in Hb concentration measurement could lead to considerable misclassification. The protocol for Hb adjustment is not clearly outlined in the DHS country-specific key indicator report, although we suspect that adjustments for ethnicity did not occur in this recent national survey [1].

We found conflicting evidence of iron deficiency based on ferritin and sTfR concentrations in all population groups, with the prevalence being consistently lower using ferritin, even after adjusting for inflammation, compared with those estimates with sTfR concentrations. We speculate that such inconsistencies may be attributed in part to genetic Hb disorders, some of which are known to significantly increase both ferritin and sTfR concentrations [37]. Certainly, Hb S variants (referred to as sickle cell trait in the heterozygous form and sickle cell disease in the homozygous form) and α-thalassemia have been detected among the Congolese population [9,10,38], which is not surprising given the frequency of these genotypes across sub-Saharan Africa [38]. Both sickle cell disease and α-thalassemia are associated with ineffective erythropoiesis that stimulates an increase in iron absorption even when iron stores are adequate, resulting in elevated levels of sTfR and ferritin [38]. Simultaneously, mild to severe anemia may occur, as a result of the negative impact on Hb concentrations, the severity depending on whether the Hb variants are homo- or heterozygous. Glucose-6-phosphate dehydrogenase deficiency, a genetic enzyme deficiency that is common among African populations [39], is another factor that may have contributed to the low prevalence of storage iron depletion reported here. Hence, both genetic Hb disorders and a genetic enzyme deficiency have the potential to confound the diagnostic accuracy of ferritin, sTfR and Hb used to identify storage iron depletion, tissue iron deficiency, as well as IDA [40,41,42]. We acknowledge that the consequences of Hb variants are likely to differ according to the type of mutation or deletion, the severity of phenotype and other co-morbidities associated with the specific Hb genotype. We conclude that more research is needed to assess the impact of Hb variants and genetic enzyme defects on ferritin and sTfR concentrations in the DRC population.

Elevated sTfR could also be a consequence of malarial infection. In our study, mean sTfR concentrations were higher among children (*p* < 0.01) and women (*p* = 0.07) in KC, where malaria was more highly prevalent, as compared to SK. In fact, a significantly higher proportion of children and women with malaria had elevated sTfR concentrations, as compared with those with no malaria. This is consistent with findings in rural Tanzanian infants, whose elevated sTfR concentrations as a result of malaria were even higher than the levels observed among the Tanzanian infants with IDA [43]. Such elevated sTfR concentrations were attributed to the hemolysis induced by malarial infection based on the positive correlation observed between sTfR concentration and parasite density in infant plasma [43]. Chronic hemolysis from malaria may have also induced increases in excretion of urinary zinc [44] and may have contributed to the high prevalence of low serum zinc concentrations reported here.

There is a tacit assumption that in areas where anemia prevalence is high, up to 50% of the anemia is due to iron deficiency [45]. Based on our findings <3% of the anemia in women was caused by iron deficiency regardless of iron biomarker used. In children, IDA was higher but never reached 20%, and only when based on elevated sTfR. Based on ferritin the highest rate of IDA was <10% in children 6–23 months in SK. In older children, rates of IDA based on ferritin were <1%. The reasons for the low rates of IDA are similar to those given for ferritin and sTfR. Although numbers are small as we determined the MCV on a sub-sample of individuals in our study, only ~33% (*n* = 7/21) of the anemic women and ~56% (*n* = 22/39) of the anemic children had a low MCV (<80 fL for women; <77 fL for children 6–35 months; and <79 fL for children 36–59 months), respectively. Of the women with microcytic anemia (low Hb and low MCV), only 17% (*n* = 1/6) had a low ferritin and similarly 17% (*n* = 1/6) had an elevated sTfR (ferritin and sTfR data was missing for *n* = 1 woman). Of the children with microcytic anemia, only 20% (*n* = 4/20) had a low ferritin and 40% (*n* = 8/20) had an elevated sTfR (ferritin and sTfR data was missing for *n* = 1 child). A low MCV often indicates iron deficiency but can also indicate the presence of thalassemia or other genetic blood disorders [37].

Folate and B12 deficiencies can also cause macrocytic anemia, characterized by a high MCV. In the sub-sample of women and children who we measured MCV, only ~9% (*n* = 2/21) of anemic women and ~13% (*n* = 5/39) of the anemic children had an elevated MCV (>96 fL for women and >86 fL for children). Likewise, the prevalence of low serum folate and B12 was <1% and <5%, respectively, in both women and children. The lack of folate deficiency is not surprising given the high consumption of beans, amaranth and other leaves, as well as cassava. B12 deficiency has been described as widespread in parts of Africa; however we found no biochemical evidence of deficiency [46]. A preliminary examination of our 24 h recall data suggests that fish consumption is high in SK which encompasses Lake Kivu and moderately high in KC, where fish may come from the Congo river or one of its tributaries. Like B12 and folate, we found almost no evidence of vitamin A deficiency based on RBP. Less than 1% of women had vitamin A deficiency, based on a low inflammation-adjusted RBP concentration in both provinces, and this was less than 10% among children in KC and 6% among in SK in both age groups. Engle-Stone *et al*. [47] suggests that the cut-offs for RBP indicative of vitamin A deficiency are population-specific. However, inflammation-adjusted serum RBP and ROH concentrations showed overall similar prevalence rates of vitamin A deficiency in a sub-sample of *n* = 48 women (<1% in both SK and KC) and *n* = 50 children 6–59 months (both < 1% in SK; 4% *vs.* 17% in KC) respectively, in our study. In a study conducted in the mid 1990’s the prevalence of deficient serum ROH concentration (<0.35 mmol/L) was 20% [48]. However, this study was conducted before widespread vitamin A supplementation. In the current study, 90% of children in both provinces received a vitamin A supplement (100,000 IU) in the previous six months. Nearly 40% of women were lactating; the national policy in the DRC is to supplement all women with 200,000 IU post-partum. Regrettably we did not record this information but according to the DHS 2013–2014 [1], the proportion of women who received vitamin A (200,000 IU) within the two months postpartum was 19% in SK and 37% in KC. In addition, red palm oil appeared to be frequently consumed, especially in KC, which depending on how it is prepared, can contain considerable amounts of pro-vitamin A carotenoids [49]. Carotenoids in red palm oil are thought to be stable unless fried several times [50].

We found that the risk of zinc deficiency based on low serum zinc concentrations was very high (ranging 52%–58% by province) among women of childbearing age. In addition, the risk of zinc deficiency among the children (ranging 20%–27% by province and age group) also exceeded levels said to be indicative of elevated public health concern [51,52]. Hence, interventions to improve zinc status among both women of childbearing age and young children in these two districts of the DRC are warranted. The etiology of zinc deficiency in DRC is uncertain. It is likely to be associated, at least in part, with inadequate intakes of dietary zinc, as reported in earlier studies in DRC [53] and elsewhere in rural Africa [44,54,55,56]. Such inadequacies may arise from low intakes of zinc and poor bioavailability of dietary zinc. In SK, the major dietary staple is cassava which has a very low zinc and phytate content [57], whereas in KC, a combination of cassava, unrefined cereals and legumes are frequently consumed [53,58]. In contrast, consumption of animal protein (e.g., meat, poultry and fish), a rich source of readily available zinc, is said to be limited in rural districts in the DRC [53]. Diets in which unrefined cereals and legumes provide a major proportion of the energy have a high content of phytate, a potent inhibitor of zinc absorption [59], suggesting that in KC, poor bioavailability may be an additional factor exacerbating the risk of zinc deficiency. Other non-dietary factors, besides inflammation [60,61], with the potential to compromise plasma zinc concentrations and thus impact prevalence estimates in these rural settings include parity [53] and possibly oral contraceptive use among women [60], tropical enteropathy among the young children [62], time of day, fasting status, time interval since the previous meal [63], and genetic Hb disorders (*i.e.,* sickle cell disease and thalassemia) [64,65]. Of these, data on time of day of sampling (*i.e.,* afternoon) and non-fasting status were collected here and appropriate cut-offs to define low serum zinc concentrations were applied accordingly [66].

Strengths of the study are that data on 744 mother-infant pairs were collected and used a probability proportionate to size sampling method to assess prevalence estimates (95%CI) for two large provinces in the DRC. We collected numerous biochemical indicators including Hb concentration, micronutrient and inflammation biomarkers, and altitude level, and adjusted concentrations for levels of inflammation, altitude, and ethnicity accordingly. Limitations are that we were not able to capture nationally representative data, or even provincial level data. The areas of SK not surveyed were more remote and often areas of civil conflict and violence and may have been at greater risk of nutritional inadequacy. In KC, we only surveyed one of five districts, and hence cannot extrapolate our findings to the whole province. The refusal rate in SK was higher than expected, but it must be remembered that this area in Eastern DRC has ongoing rebel and military conflict and trust of outsiders is low. We did not test individuals for genetic Hb disorders, genetic enzymatic disorders, or hemolytic conditions (with the exception of malaria) that may have contributed to increased ferritin and/or sTfR concentrations. Menstrual blood loss is also a strong determinant of iron stores in women of reproductive age [67] that was not captured in our analysis. Further, we did not collect data on oral contraceptive use; however, in other areas of the DRC the prevalence of women using oral contraceptives has been very low, and in one study it was reported that less than 10% of women of reproductive age used oral contraceptives [68].

## 5. Conclusions

Anemia prevalence varied widely by Hb adjustments for altitude and ethnicity among women of childbearing age and children 6–59 months in SK and KC provinces. The adjustment of Hb for ethnic origin is controversial and recommendations vary among organizations and publications. As we have shown in our study, there are substantial implications of this ethnic adjustment as it significantly impacts anemia prevalence rates in African populations. We conclude that zinc deficiency but seemingly not storage iron depletion (low ferritin) was prevalent and that IDA was apparently very low in our study. We urge caution in the interpretation of both sTfR and ferritin because of the high burden of infection and inflammation, as well as the potentially high prevalence of genetic Hb disorders. The high risk of zinc deficiency suspected among women and young children in the DRC warrants urgent attention.

## Figures and Tables

**Table 1:** Household characteristics of the study population in two provinces in the Democratic Republic of the Congo.

	South Kivu	Kongo Central
Total, *n* (%)	444 (59.7)	300 (40.3)
Household size, mean ± SD	6.6 ± 2.6	5.7 ± 2.3
Mother’s education level, *n* (%)		
No schooling	214/444 (48.2)	29/299 (9.7)
Primary school	139/444 (31.3)	141/299 (47.2)
Secondary	87/444 (19.6)	128/299 (42.8)
Tertiary/higher education	2/444 (0.5)	1/299 (0.3)
Other	2/444 (0.5)	0/299 (0)
Material of household roof, *n* (%)		
Leaf	118/443 (26.6)	186/300 (62.0)
Tiles	18/443 (4.1)	19/300 (6.3)
Metal/tin	306/443 (69.1)	95/300 (31.7)
Other	1/443 (0.2)	0/300 (0)
Main source of household fuel, *n* (%)		
Electricity	11/443 (2.5)	26/300 (8.7)
Liquified petroleum gas	2/443 (0.5)	0/300 (0)
Natural gas	15/443 (3.4)	2/300 (0.7)
Kerosene	86/443 (19.4)	79/300 (26.3)
Charcoal	325/443 (73.4)	193/300 (64.3)
Other	4/443 (0.9)	0/300 (0)
Main source of drinking water, *n* (%)		
Piped water	254/441 (57.6)	34/300 (11.3)
Open well	9/441 (2.0)	1/300 (0.3)
Covered well/borehole	29/441 (6.6)	15/300 (5.0)
Surface water (spring, river, or pond)	101/441 (22.9)	248/300 (82.7)
Rainwater	22/441 (5.0)	2/300 (0.7)
Other	26/441 (5.9)	0/300 (0)
Type of household toilet, *n* (%)		
No facility (bush or field)	16/443 (3.6)	45/299 (15.1)
Flush toilet	6/443 (1.4)	14/299 (4.7)
Pit latrine	420/443 (94.8)	239/299 (79.9)
Bucket	1/443 (0.2)	1/299 (0.3)
Does your household have a bednet, *n* (%)		
Yes	281/444 (63.3)	205/300 (68.3)
Has the child received a vitamin A capsule in the past 6 months, *n* (%)		
Yes	397/444 (89.4)	279/299 (93.3)
Has the child received a deworming tablet in the last 6 months, *n* (%)		
Yes	368/444 (82.8)	232/300 (77.3)
Has the child received iron syrup or tablets in the last 3 months, *n* (%)		
Yes	36/442 (8.1)	30/298 (10.1)

**Table 2:** Mean ± SEM age and concentration of micronutrients, hemoglobin, and inflammation biomarkers among women of childbearing age (*n*= 713–743 women) by province ^1^.

	South Kivu	Kongo Central	*p*
Total, *n* (%)	444 (59.7)	300 (40.3)	NA
Age, year	29.2 ± 0.4	29.9 ± 0.4	0.2
Micronutrients			
Vitamin B12, pmol/L	528 ± 11	684 ± 16	<0.00001
RBP (unadjusted), µmol/L	1.89 ± 0.03	1.70 ± 0.04	<0.00001
RBP (adjusted) ^2^, µmol/L	1.95 ± 0.03	1.75 ± 0.04	<0.00001
Zinc (unadjusted), µmol/L	8.9 ± 0.1	9.0 ± 0.3	0.9
Zinc (adjusted) ^3^, µmol/L	9.1 ± 0.8	9.2 ± 0.3	0.8
Folate, nmol/L	38 ± 0.9	22 ± 0.8	<0.00001
Ferritin (unadjusted), µg/L	80 ± 3	61 ± 2	<0.00001
Ferritin (adjusted) ^2^, µg/L	72 ± 2	54 ± 2	<0.00001
sTfR, mg/L	6.8 ± 0.1	7.3 ± 0.2	0.07
Hb, g/L			
Unadjusted Hb	135 ± 1	127 ± 1	<0.00001
Hb adjusted for altitude	131 ± 1	126 ± 1	<0.00001
Hb adjusted for ethnicity	145 ± 1	137 ± 1	<0.00001
Hb adjusted for altitude and ethnicity	141 ± 1	136 ± 1	<0.00001
Inflammation Biomarkers			
Acute, CRP, mg/L	2.09 ± 0.28	2.28 ± 0.31	0.6
Chronic, AGP, g/L	0.83 ± 0.02	0.87 ± 0.03	0.3

^1^ AGP, α-1 acid glycoprotein; CRP, C-reactive protein; Hb, hemoglobin; NA, not applicable; RBP, retinol binding protein (vitamin A status); SEM, standard error of the mean; sTfR, soluble transferrin receptor. Mean concentrations between provinces were compared using *t*-tests; ^2^ Values were adjusted for inflammation using correction factors proposed by Thurnham *et al*. [24,25]; ^3^ Values were adjusted for inflammation using study-generated correction factors.

**Table 3:** Mean ± SEM age and concentration of micronutrients, hemoglobin, and inflammation biomarkers among children 6–23 and 24–59 months (*n* = 676–741 children) by province ^1^.

	6–23 Months (*n* = 286)			24–59 Months (*n* = 458)		
	South Kivu	Kongo Central	*P*	South Kivu	Kongo Central	*p*
Total, *n* (%)	158 (55.2)	128 (44.8)	NA	286 (62.4)	172 (37.6)	NA
Age, month	14.2 ± 0.4	14.9 ± 0.4	0.2	37.3 ± 0.5	38.0 ± 0.7	0.4
Micronutrients						
Vitamin B12, pmol/L	370 ± 15	443 ± 18	0.002	487 ± 14	630 ± 22	<0.00001
RBP (unadjusted), µmol/L	1.10 ± 0.03	0.93 ± 0.03	0.0001	1.08 ± 0.02	0.92 ± 0.02	<0.00001
RBP (adjusted) ^2^, µmol/L	1.20 ± 0.03	1.05 ± 0.03	0.0008	1.16 ± 0.02	1.04 ± 0.02	0.0003
Zinc (unadjusted), µmol/L	9.5 ± 0.2	9.6 ± 0.2	0.7	9.5 ± 0.1	9.2 ± 0.2	0.2
Zinc (adjusted) ^3^, µmol/L	10.1 ± 0.2	10.3 ± 0.2	0.5	10.0 ± 0.1	9.9 ± 0.2	0.8
Folate, nmol/L	41 ± 1	27 ± 1	<0.00001	43 ± 1	29 ± 1	<0.00001
Ferritin (unadjusted), µg/L	43 ± 4	86 ± 8.3	<0.00001	71 ± 3	126 ± 6	<0.00001
Ferritin (adjusted) ^2^, µg/L	32 ± 3	55 ± 5	<0.00001	55 ± 2	84 ± 4	<0.00001
sTfR, mg/L	10.5 ± 0.5	12.7 ± 0.6	0.003	7.8 ± 0.2	10.3 ± 0.4	<0.00001
Hb, g/L						
Unadjusted Hb	111 ± 1	113 ± 2	0.4	118 ± 1	113 ± 1	0.0005
Hb adjusted for altitude	107 ± 1	112 ± 2	0.003	113 ± 1	112 ± 1	0.6
Hb adjusted for ethnicity	121 ± 1	123 ± 2	0.4	128 ± 1	123 ± 1	0.0005
Hb adjusted for altitude and ethnicity	117 ± 1	123 ± 2	0.003	123 ± 1	122 ± 1	0.6
Inflammation Biomarkers						
Acute, CRP, mg/L	4.98 ± 0.83	9.46 ± 1.27	0.003	3.81 ± 0.51	10.67 ± 1.12	<0.00001
Chronic, AGP, g/L	1.46 ± 0.07	1.68 ± 0.08	0.05	1.51 ± 0.05	1.75 ± 0.07	0.008

^1^ AGP, α-1 acid glycoprotein; CRP, C-reactive protein; Hb, hemoglobin; NA, not applicable; RBP, retinol binding protein (vitamin A status); SEM, standard error of the mean; sTfR, soluble transferrin receptor. Mean concentrations between provinces for each separate age group (6–23 or 24–59) were compared using *t*-tests; ^2^ Values were adjusted for inflammation using correction factors proposed by Thurnham *et al*. [24,25]; ^3^ Values were adjusted for inflammation using study-generated correction factors.

**Table 4:** Body mass index (BMI) and prevalence of micronutrient deficiencies, anemia, iron deficiency, inflammation and malaria among women of childbearing age (*n* = 696–741 women) by province ^1^.

	South Kivu	Kongo Central
Total, *n* (%)	444 (59.7)	300 (40.3)
BMI, kg/m^2^, *n* (%)		
Underweight, <18.5	13 (3.0)	45 (15.0)
Normal, 18.5–24.9	316 (73.5)	228 (76.0)
Overweight, 25.0–29.9	91 (21.2)	22 (7.3)
Obese, ≥30.0	10 (2.3)	5 (1.7)
Micronutrient Deficiencies, % (95%CI)		
Vitamin B12, <150 pmol/L	0.5 (0.1, 1.6)	0.7 (0.08, 2.6)
RBP (unadjusted), <0.7 µmol/L	0.2 (0.0, 1.2)	1.3 (0.36, 3.4)
RBP (adjusted) ^2^, <0.7 µmol/L	0 (0, 0.8) *	0.7 (0.1, 2.4)
Zinc (unadjusted), <9.0 µmol/L	55.1 (50.3, 59.8)	60.0 (54.2, 65.6)
Zinc (adjusted) ^3^, <9.0 µmol/L	51.8 (47.0, 56.6)	57.7 (51.9, 63.3)
Folate, <6.8 nmol/L	0.7 (0.1, 2.0)	1.0 (0.2, 3.0)
Ferritin (unadjusted), <15 µg/L	3.9 (2.3, 6.1)	4.7 (2.6, 7.7)
Ferritin (adjusted) ^2^, <15 µg/L	5.4 (3.5, 8.0)	5.3 (3.1, 8.5)
sTfR, >8.3 mg/L	17.7 (14.2, 21.6)	20.7 (16.2, 25.7)
Anemia, Hb < 120 g/L, % (95%CI)		
Unadjusted Hb	10.2 (7.5, 13.4)	29.2 (24.1, 34.7)
Hb adjusted for altitude	16.5 (13.2, 20.3)	31.9 (26.7, 37.5)
Hb adjusted for ethnicity	3.4 (1.9, 5.5)	9.4 (6.3, 13.2)
Hb adjusted for altitude and ethnicity	5.9 (3.9, 8.5)	10.4 (7.2, 14.4)
Iron Deficiency Anemia, % (95%CI)		
Hb < 120 g/L and ferritin < 15 µg/L ^2^	0.9 (0.2, 2.3)	0.7 (0.1, 2.4)
Hb < 120 g/L and sTfR > 8.3 mg/L	2.7 (1.4, 4.7)	2.3 (0.9, 4.8)
Inflammation Biomarkers, % (95%CI)		
Acute, CRP, >5 mg/L	6.3 (4.3, 9.0)	9.7 (6.7, 13.6)
Chronic, AGP, >1 g/L	22.7 (18.8, 26.9)	23.7 (19.0, 28.9)
Malaria Infection, % (95%CI)	1.8 (0.7, 3.6)	7.3 (4.7, 10.9)

^1^ AGP, α-1 acid glycoprotein; CRP, C-reactive protein; Hb, hemoglobin; RBP, retinol binding protein (vitamin A status); sTfR, soluble transferrin receptor; ^2^ Values were adjusted for inflammation using correction factors proposed by Thurnham *et al*. [24,25]; ^3^ Values were adjusted for inflammation using study-generated correction factors; * Values present one-sided 97.5% CI (rather than two-sided 95%CI) due to very low prevalence rates.

**Table 5:** Prevalence of micronutrient deficiencies, anemia, iron deficiency, inflammation and malaria among children 6–23 and 24–59 months (*n* = 676–744) by province ^1^.

	6–23 Months		24–59 Months	
	South Kivu	Kongo Central	South Kivu	Kongo Central
Total, *n* (%)	158 (55.2)	128 (44.8)	286 (62.4)	172 (37.6)
Micronutrient Deficiencies, % (95%CI)				
Vitamin B12, <150 pmol/L	4.9 (2.0, 9.8)	1.8 (0.2, 6.5)	3.0 (1.3, 5.9)	0.0 (0.0, 2.3) *
RBP (unadjusted), <0.7 µmol/L	6.5 (3.0, 11.9)	19.0 (12.4, 27.1)	12.0 (8.3, 16.6)	24.3 (18.0, 31.4)
RBP (adjusted) ^2^, <0.7 µmol/L	1.3 (0.2, 4.5)	9.4 (4.9, 15.8)	5.6 (3.2, 8.9)	9.9 (5.9, 15.4)
Zinc (unadjusted), <8.7 µmol/L	36.1 (28.2, 44.5)	31.0 (22.8, 40.3)	34.7 (29.0, 40.7)	38.7 (31.1, 46.6)
Zinc (adjusted) ^3^, <8.7 µmol/L	23.1 (16.3, 31.2)	20.0 (13.1, 28.4)	24.8 (19.6, 30.6)	27.2 (20.5, 34.7)
Folate, <6.8 nmol/L	0 (0.0, 2.5) *	0 (0.0, 3.3) *	0 (0.0, 1.4) *	0 (0.0, 2.3) *
Ferritin (unadjusted), <12 µg/L	14.3 (9.0, 21.3)	9.9 (5.2, 16.7)	4.3 (2.1, 7.5)	0.6 (0.0, 3.3)
Ferritin (adjusted) ^2^, <12 µg/L	23.0 (16.3, 30.9)	15.7 (9.7, 23.4)	5.4 (3.0, 8.9)	0.6 (0.0, 3.3)
sTfR, >8.3 mg/L	51.1 (42.5, 59.6)	74.4 (65.6, 81.9)	23.2 (18.2, 28.9)	58.0 (50.2, 65.5)
Anemia, Hb < 110 g/L, % (95%CI)				
Unadjusted Hb	45.2 (37.3, 53.4)	41.4 (32.8, 50.4)	24.2 (19.4, 29.6)	42.7 (35.2, 50.5)
Hb adjusted for altitude	58.6 (50.5, 66.4)	42.2 (33.5, 51.2)	35.4 (29.9, 41.3)	44.4 (36.9, 52.2)
Hb adjusted for ethnicity	18.5 (12.7, 25.4)	21.1 (14.3, 29.2)	8.4 (5.5, 12.3)	22.8 (16.7, 29.8)
Hb adjusted for altitude and Ethnicity	26.1 (19.4, 33.7)	25.0 (17.8, 33.4)	14.0 (10.2, 18.6)	22.8 (16.7, 29.8)
Iron Deficiency Anemia ^2,4^, % (95%CI)				
Hb < 110 g/L and ferritin < 12 µg/L	9.4 (5.1, 15.6)	2.5 (0.5, 7.1)	0.4 (0.0, 2.1)	0.0 (0.0, 2.2) *
Hb < 110 g/L and sTfR > 8.3 mg/L	17.4 (11.5, 24.8)	20.7 (13.8, 29.0)	5.1 (2.7, 8.5)	18.5 (12.9, 25.2)
Inflammation Biomarkers, % (95%CI)				
Acute, CRP, >5 mg/L	22.3 (15.7, 30.1)	42.1 (33.2, 51.5)	16.7 (12.3, 21.8)	42.6 (35.0, 50.4)
Chronic, AGP, >1 g/L	64.7 (56.2, 72.7)	71.9 (63.0, 79.7)	61.2 (55.0, 67.2)	71.0 (63.5, 77.7)
Malaria Infection, % (95%CI)	2.2 (0.5, 6.3)	33.6 (25.5, 42.5)	4.3 (2.1, 7.5)	44.2 (36.6, 51.9)

^1^AGP, α-1 acid glycoprotein; CRP, C-reactive protein; Hb, hemoglobin; RBP, retinol binding protein (vitamin A status); sTfR, soluble transferrin receptor; ^2^ Values were adjusted for inflammation using correction factors proposed by Thurnham *et al*. [24,25]; ^3^ Values were adjusted for inflammation using study-generated correction factors; ^4^ Iron deficiency anemia was calculated based on Hb concentration adjusted for both altitude and ethnicity; * Values present one-sided 97.5% CI (rather than two-sided 95%CI) due to very low prevalence rates.

**Table 6:** Proportion of children 6–23 and 24–59 months (*n* = 702) as underweight, wasted, stunted, and with low mid-upper arm circumference by province ^1^.

	South Kivu	Kongo Central	All Children
Total, *n* (%)	444 (59.7)	400 (40.3)	744 (100)
Underweight ^2^, WAZ < −2 SD			
6–23 months	25 (17.0)	17 (14.2)	42 (15.7)
24–59 months	67 (26.2)	43 (25.9)	110 (26.1)
Wasted ^3^, WHZ < −2 SD			
6–23 months	10 (6.8)	7 (5.8)	17 (6.4)
24–59 months	9 (3.6)	8 (5.2)	17 (4.2)
Stunted ^4^, HAZ < −2 SD			
6–23 months	68 (46.3)	36 (30.0)	104 (39.0)
24–59 months	168 (67.2)	82 (53.3)	250 (61.9)

^1^ Total *n* = 702 after exclusion of *n* = 42 children with extreme outliers. HAZ, height-for-age z-score; MUAC, mid-upper arm circumference; SD, standard deviation; WAZ, weight-for-age z-score; WHZ, weight-for-height z-score; ^2^ *n* = 689, excluding extreme outliers (WAZ ≤ −6 or ≥5) and *n* = 13 missing values; ^3^ *n* = 671, excluding extreme outliers (WHZ ≤ −5 or ≥5) and *n* = 31 missing values; ^4^ *n* = 671, excluding extreme outliers (HAZ ≤ −6 or ≥6) and *n* = 31 missing values.
